# The Emergence of Rotavirus G12 and the Prevalence of Enteric Viruses in Hospitalized Pediatric Diarrheal Patients in Southern Vietnam

**DOI:** 10.4269/ajtmh.2011.11-0364

**Published:** 2011-10-01

**Authors:** Phan Vu Tra My, Maia A. Rabaa, Ha Vinh, Edward C. Holmes, Nguyen Van Minh Hoang, Nguyen Thanh Vinh, Le Thi Phuong, Nguyen Thi Tham, Phan Van Be Bay, James I. Campbell, Jeremy Farrar, Stephen Baker

**Affiliations:** Hospital for Tropical Diseases, Wellcome Trust Major Overseas Programme, Oxford University Clinical Research Unit, Ho Chi Minh City, Vietnam; Center for Infectious Disease Dynamics, Department of Biology, Pennsylvania State University, University Park, Pennsylvania; Hospital for Tropical Diseases, Ho Chi Minh City, Vietnam; Fogarty International Center, National Institutes of Health, Bethesda, Maryland; Dong Thap Provincial Hospital, Dong Thap, Vietnam; Centre for Tropical Diseases, University of Oxford, Oxford, United Kingdom

## Abstract

Diarrhea is a major cause of childhood morbidity and mortality in developing countries, and the majority of infections are of viral etiology. We aimed to compare the etiological prevalence of the major enteric viruses in an urban and a rural setting in southern Vietnam. We simultaneously screened fecal specimens from 362 children in Ho Chi Minh City and Dong Thap province that were hospitalized with acute diarrhea over a 1-month-long period for four viral gastrointestinal pathogens. Rotavirus was the most common pathogen identified, but there was a differential prevalence of rotavirus and norovirus between the urban and rural locations. Furthermore, rotavirus genotyping and phylogenetic analysis again differentiated the genotypes by the sampling location. Our data show a disproportional distribution of enteric viral pathogens in urban and rural locations, and we provide evidence of continual importation of new rotavirus strains into southern Vietnam and report the emergence of rotavirus genotype G12.

## Introduction

Diarrhea is the second most common cause of childhood mortality worldwide, estimated to be responsible for 1.76 million deaths annually between 2000 and 2003 and 1.87 million deaths in children under the age of 5 years in 2004.^1^–^3^ It is young children and infants in unindustrialized and industrializing countries that are disproportionately affected, and in such locations, diarrheal infections are often severe, frequently requiring hospitalization.^4^ The etiological agents of diarrhea are numerous, including multiple viral, bacterial, and parasitic pathogens. However, it is viruses that are responsible for the vast burden of morbidity and mortality, causing up to 40% of all severe diarrhea cases in developing countries.^5^ The most prevalent viruses causing endemic childhood gastroenteritis are rotavirus, norovirus, enteric adenovirus, and astrovirus.^6^ Rotavirus is the dominant agent of viral diarrhea and is the suspected etiological agent in 39% and 45% of all hospital admissions related to diarrhea globally and in Asia, respectively.^7^,^8^ In Vietnam, rotavirus is estimated to be responsible for between 44% and 67.4% of all childhood diarrheal infections requiring hospitalization.^9^–^11^

Rotavirus has a genome comprised of 11 segments of double-stranded RNA (dsRNA) encoding six structural proteins and six non-structural proteins.^12^ It is genetic heterogeneity in two of the structural regions that encode the viral capsid proteins, VP7 and VP4, that permit the differentiation of individual rotavirus strains.^12^ Sequencing of the VP7 region (glycoprotein) defines the G type, and VP4 region sequencing (protease-sensitive protein) determines the P type. To date, 19 G and 28 P genotypes have been described, 10 and 11 of which, respectively, have been isolated from humans.^12^,^13^ Strain G1P[8] is the most frequent rotavirus A GP combination isolated from symptomatic humans worldwide.^14^

Transmission and distribution of rotavirus A is complex and influenced by multiple social, demographic, and environmental factors.^15^,^16^ Vietnam is a typical industrializing country where the agents of infectious diseases are changing rapidly.^17^ Existing data on viral gastrointestinal pathogens are available from sentinel surveillance in Ho Chi Minh City (HCMC) between 1998 and 2007.^18^–^24^ However, because detection and identification of enteric pathogens are not performed routinely in hospitals, little is known about the prevalence of viral diarrheal pathogens and the strains that circulate in different geographic and demographic locations in Vietnam. We aimed to investigate the distribution of norovirus, enteric adenovirus, astrovirus, and rotavirus genotypes causing diarrhea in hospitalized children in distinct urban and rural locations in southern Vietnam.

## Materials and Methods

### Study sites and population.

Verbal informed consent was obtained from the parents or legal guardians of minors enrolled in this study. This work was approved by the institutional ethical review boards of the Hospital for Tropical Diseases, HCMC and Dong Thap Provincial Hospital, Dong Thap. Patient recruitment was performed over one calendar month from November 1, 2008 to November 30, 2008 at two hospitals, pediatric ward B at the Hospital for Tropical Diseases (HTD) in HCMC and pediatric infections ward at Dong Thap Provincial Hospital (DTPH) in Dong Thap (DT) province. DTPH is 154 km away from HCMC and located within the Mekong Delta region of southern Vietnam; it is a rural location, with a lower population density than HCMC. We enrolled all pediatric (under the age of 15 years) patients who had been hospitalized at HTD or DTPH because of acute watery diarrhea (defined as three or more loose stools within a 24-hour period) without any additional underlying complications, such as febrile convulsions, extensive dehydration, or stool-containing blood or mucus. The age of each patient was recorded, and a stool specimen from each patient was collected in a sterile container on the day of admission and was stored at −20°C.

### RNA extraction and virus detection.

Total viral RNA was extracted from 10% (in phosphate-buffered saline [PBS]) fecal specimens using the QIAamp viral RNA Mini kit (QIAGEN, Hilden, Germany) according to the manufacturer's recommendations. RNA preparations were converted to complementary DNA (cDNA) by reverse transcription (RT), and an aliquot of RNA of each sample was stored at −80°C until required. For RT, extracted RNA was reverse-transcribed by SuperScript Reverse Transcriptase III and RNase Inhibitor (Invitrogen) combined with a random hexamer (Roche Diagnostics, United Kingdom) according to the manufacturer's instructions. The resulting cDNA were stored at −80°C.

All stool samples were screened for rotavirus A, norovirus (genogroups I and II), enteric adenovirus, and astrovirus using IDEIA^TM^ direct antigen detection kits according to the manufacturer's instructions (Oxoid; Thermo Fisher Scientific, United Kingdom). Rotavirus outer capsid genes (VP7 and VP4) detection was performed by RT-polymerase chain reaction (PCR) in stool specimens that were positive for EIA rotavirus A. Briefly, viral cDNA was subjected to RT-PCR to amplify the VP7 and VP4 genes using primers and amplification conditions as previously described.^25^ Amplification of VP7 and VP4 regions was performed individually, and PCR reactions were predicted to generate amplicons of 881 and 663 bp, respectively. PCR amplicons were visualized on 2% agarose gels under ultraviolet (UV) light after staining with 3% ethidium bromide.

### Sequencing, genotype determination, and phylogenetic analysis.

PCR amplicons from successful VP7 and VP4 PCR amplifications were DNA sequenced using the amplification primers. PCR amplicons were purified using the QIAquick PCR purification kit (QIAGEN, Germany), and DNA concentrations were determined using a NanoDrop ND-1000 spectrophotometer (Thermo Fisher Scientific, United Kingdom). Direct DNA sequencing was performed using the BigDye Terminator Cycle Sequencing kit (Applied Biosystems) according to the manufacturer's recommendations. All DNA sequences were generated using an ABI Prism 3130xl Genetic Analyzer (Applied Biosystems), and the resulting DNA sequences were assembled using DNA Baser Sequence Assembler v3.0.17 (Heracle Biosoft, Pitesti, Romania).

The resulting VP4 and VP7 sequences (Genbank accession numbers: VP4, FR820957–FR821065; VP7, FR822209–FR822321) were compared with other corresponding genotype sequences using BLASTn (https://blast.ncbi.nlm.nih.gov/ Blast.cgi) for genotype determination. Coding sequences were manually aligned using Se-AL v2.0a11 (http://tree.bio.ed.ac.uk/software/), and additional sequences were trimmed to correspond with our sequences to maximize sequence homology. Maximum likelihood trees for each of the genotypes were inferred using RAxML v 7.0.4^26^ employing the general time-reversible model of nucleotide substitution with a γ-distribution of among-site rate variation (GTR+Γ), which was determined using jModelTest.^27^ One thousand bootstrap replicates were used as implemented in a rapid bootstrap algorithm available in RAxML. The resulting trees were visualized in FigTree v1.3.1 (http://tree.bio.ed.ac.uk/software/figtree/), and genetic distances were estimated using HyPhy v2.0 (http://www.datam0nk3y.org/hyphy/doku.php).^28^ All statistical analyses were performed in R version 2.9.0 (The R foundation for Statistical Computing); *P* values < 0.05 were considered statistically significant.

## Results

### Enteric virus prevalence.

A total of 362 children from the two locations (252 from HCMC and 110 from DT) with acute gastroenteritis were concurrently enrolled in the 1-month-long study. We were able to detect rotavirus A, norovirus, adenovirus, or astrovirus using EIA in 195 samples (53.9%), and eight children had more than one viral pathogen in their stool (Table 1). In the 252 samples originating from HCMC, 75 (29.8%) were positive for group A rotavirus, and 34 (13.5%) were positive for norovirus (Table 1). In the DT samples, 72 (65.5%) were positive for group A rotavirus, and 4 (3.6%) were positive for norovirus. Enteric adenovirus and astrovirus collectively comprised only a small proportion of all the acute diarrheal cases in HCMC (2.4% and 2.8%, respectively); this finding was also the case in DT (2.7% and 1.8%, respectively).

The age distribution of all the hospitalized patients with diarrhea was similar in both locations, with a mean of 15.8 months (median = 13 months, range = 2–96 months) in HCMC and a mean of 15.3 months (median = 10 months, range = 1.5–156 months) in DT. The preponderance of patients were less than 24 months of age (90.6% in HCMC and 94.9% in DT), and the most common 6-month age group was between 7 and 12 months. This peak age group was comprised of 43.6% (51/117) of the positive samples from HCMC and 48.7% (38/78) of the positive samples from DT. We found that the prevalence of viral positive samples in cases from HCMC was significantly lower in those children younger than 6 months and older than 24 months of age compared with those children in the intermediate age group (*P* < 0.001, χ^2^ test). In DT, the prevalence of viral diarrhea was lower in patients older than 18 months of age compared with children aged between 0 and 18 months (*P* < 0.001, χ^2^ test). Furthermore, the proportion of rotavirus infections in patients under the age of 6 months from DT was significantly higher than the corresponding age group from HCMC (*P* = 0.0289, χ^2^ test). Conversely, in the group consisting of children aged from 19 to 24 months, the proportion of rotavirus infections was significantly greater in HCMC than DT (*P* = 0.009457, χ^2^ test).

### Distribution of rotavirus genotypes.

**Figure 1.**
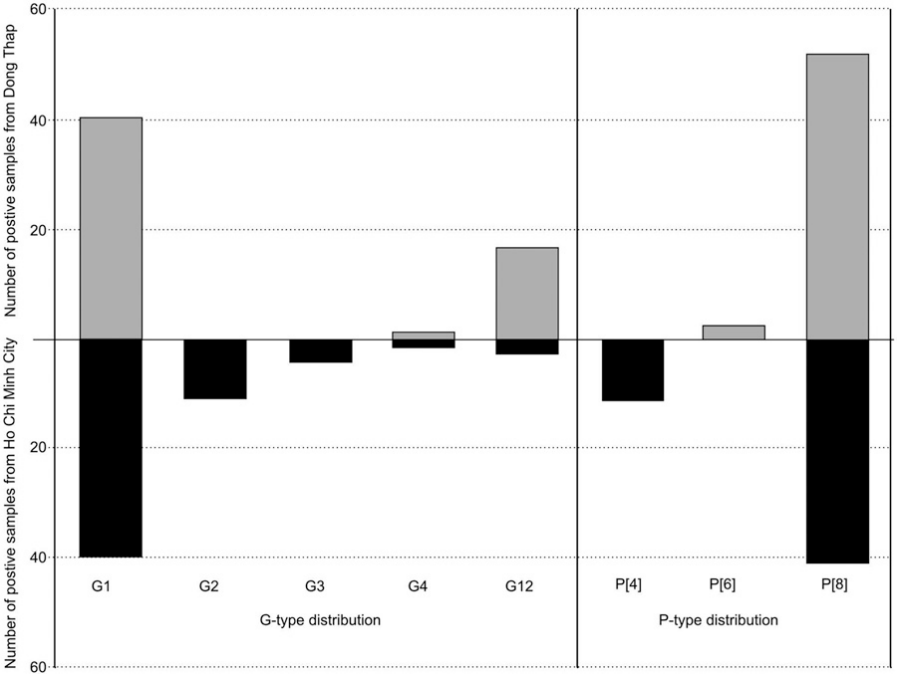
The distribution of rotavirus G and P types detected in the stools of children with diarrhea in Ho Chi Minh City and Dong Thap. The graph shows the distribution of rotavirus G types 1, 2, 3, 4, and 12 and P types P[4], P[6], and P[8] in 118 and 109 VP7 and VP4 PCR amplification-positive samples, respectively. The graph is subdivided into G (*N* = 60) and P types (*N* = 56) from DT (upper, grey) and G (*N* = 58) and P types (*N* = 53) from HCMC (lower, black).

All stool samples were subjected to RT-PCR for the VP4 and VP7 regions of rotavirus A. None of the samples that were negative for rotavirus using EIA were positive by PCR, and 133 of 157 samples of the positive EIA samples gave a PCR amplicon for one or both of the loci (PCR compared with EIA: sensitivity, 90%; specificity, 100%). All positive VP4 and VP7 PCR amplicons were DNA-sequenced; 118 of 133 amplicons produced a sequence consistent with the VP4 region, and 109 of 133 amplicons produced a sequence consistent with the VP7 region. We found that genotype G1 was the dominant circulating G type, comprising 82 of 118 (69.5%) of all typeable G types (Figure 1). The novel emerging genotype, G12, was the second most common G type, representing 16.1% (19/118) of all G types. Other less common G genotypes, including G2, G3, and G4, were also detected in small proportions in the remainder of the positive samples (Figure 1). Genotype P[8] was the most prevalent typeable P genotype, accounting for 88.1% (96/109) of all P amplicons, followed by P[4] (10.1%, 11/109) and P[6] (1.8%, 2/109).

The most common GP combination was G1P[8], comprising 78.5% (73/93) of all samples that were positive for both amplification targets. Other globally diffuse GP genotypes, including G2P[4] and G3P[8], were also detected but again, in a limited number of samples (10.8%, 10/93; 2.2%, 2/93, respectively) (Figure 1). The overall distribution of G and P types differed substantially between the two locations. Genotypes G2 and G3 were present in several rotavirus positive samples in HCMC, but neither was identified in positive amplifications from DT. Genotype G12 was identified in both locations but was more prevalent in DT than in HCMC, representing 28.3% and 3.4% of all G types in these locations, respectively (*P* = 0.0016, χ^2^ test). Furthermore, P[4] was identified in 20.8% of the P-type samples in HCMC but was not detected in DT, and P[6] was detected only in samples originating from DT.

### Phylogenetic analysis of rotavirus sequences.

**Figure 2.**
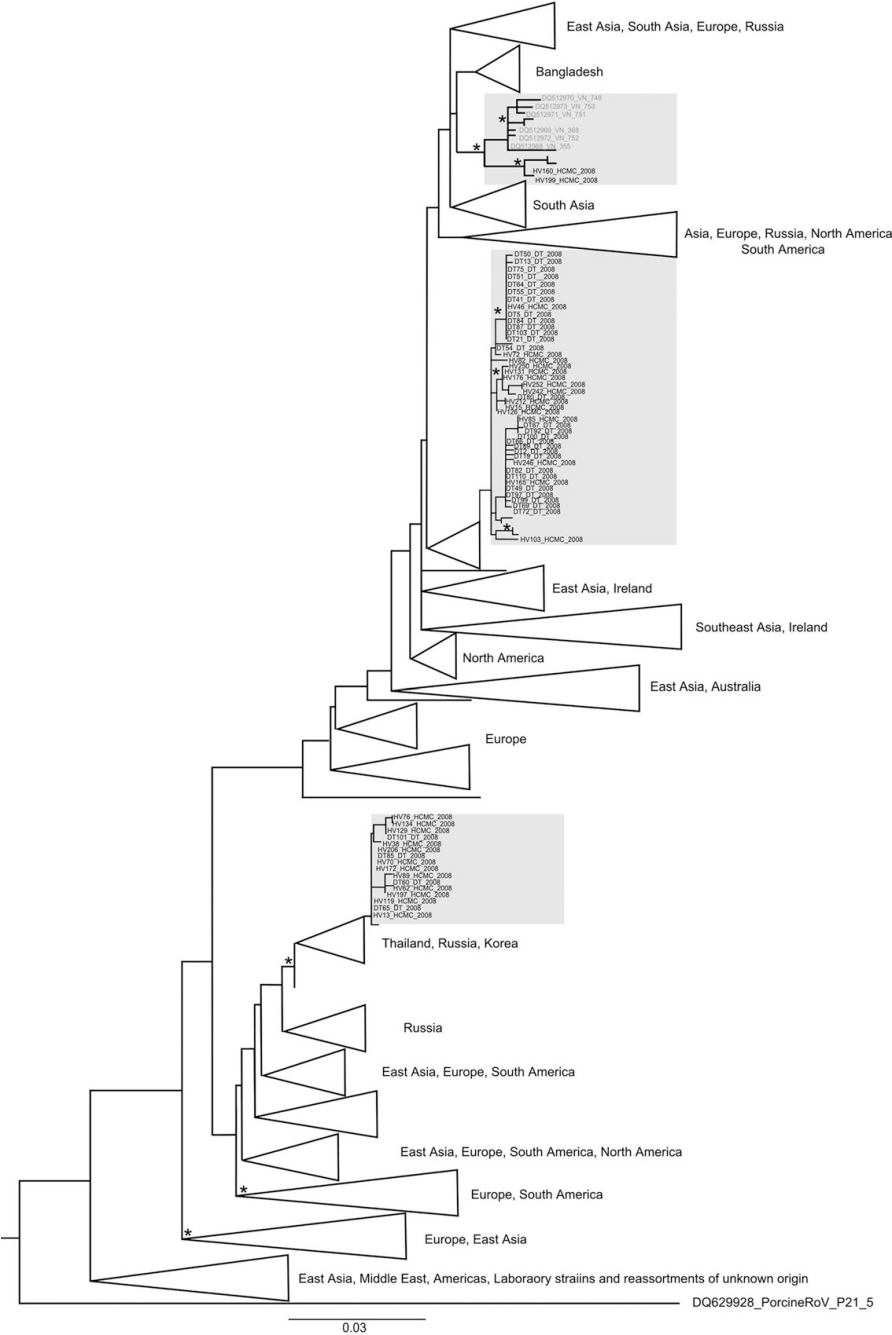
Phylogenetic tree of 81 rotavirus G1 sequences from Ho Chi Minh City and Dong Thap combined with representative global rotavirus G1 sequences. Maximum likelihood phylogenetic tree was constructed from G1 sequences from the amplification and sequencing of the VP7 gene. Sequences generated from this study are indicated in black. HCMC indicates samples from Ho Chi Minh City, and DT indicates samples from Dong Thap. Vietnamese isolates from previous studies are highlighted in grey. The tree is midpoint-rooted, with all horizontal branch lengths drawn to the scale of a nucleotide substitution per site. Bootstrap values > 85% are indicated by asterisks, and triangles represent compressed regions of the tree.

**Figure 3.**
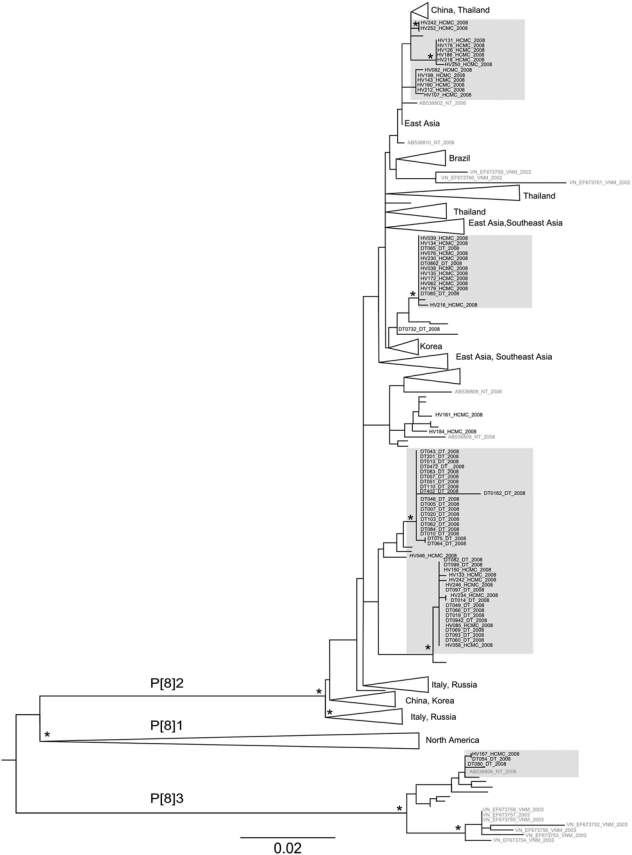
Phylogenetic tree of 96 rotavirus P[8] sequences from Ho Chi Minh City and Dong Thap combined with representative global rotavirus P[8] sequences. Maximum likelihood phylogenetic tree [VP4 gene] constructed from P[8] sequences and representative global sequences of rotavirus P[8] type. Tree rooting, bootstrap values, branch lengths, and font correspond to those factors presented in Figure 2.

**Figure 4.**
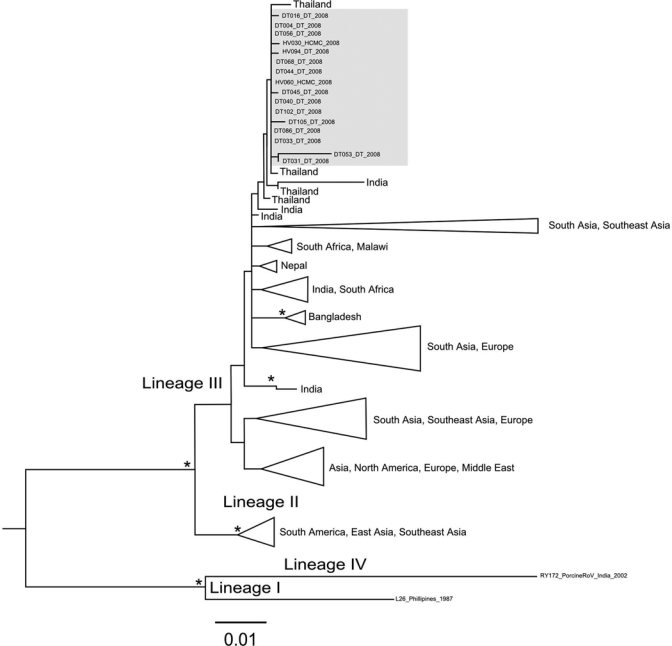
Phylogenetic tree of 19 rotavirus G12 sequences from Ho Chi Minh City and Dong Thap combined with representative global rotavirus G12 sequences. Maximum likelihood phylogenetic tree [VP7 gene] constructed from G12 sequences and representative global sequences of rotavirus G12. Tree rooting, bootstrap values, branch lengths, and font correspond to those factors presented in Figure 2.

We performed phylogenetic analyses on the G1, G12, and P[8] sequences produced from the VP7 and VP4 amplifications, comparing them with additional global sequences available in public databases. The G1 rotavirus sequences from DT and HCMC exhibited extensive genetic diversity [maximum genetic distance (uncorrected) = 0.0774] and could be differentiated into three distinct lineages (Figure 2). We could identify a significant phylogenetic association between the DT and HCMC G1 sequences, signifying the circulation of closely related rotavirus strains in these two locations. Comparing these G1 data with previous rotavirus sequence data originating in Vietnam, one lineage showed a close phylogenetic relationship to rotaviruses also isolated in HCMC, indicating local persistence of this particular lineage (Figure 2).^29^ The distribution of the P[8]-type sequences was comparable with the distribution observed throughout the G1 sequences, exhibiting extensive genetic diversity (maximum genetic distance [uncorrected] = 0.159) (Figure 3). The P[8] sequences could be differentiated into four phylogenetically distinct clusters that seem to be closely related to sequences from other regions around Asia (Figure 3). Sequences from the G12 lineage, which were primarily detected in samples from DT, were closely related to Thai and Indian sequences (Figure 4). However, less overall diversity was detected among the G12 genotype sequences (maximum genetic distance [uncorrected] = 0.0130), with only one major lineage identified (Figure 4), signifying potential recent introduction of this variant.

## Discussion

Enteric viruses are a predominant cause of acute childhood gastroenteritis in Vietnam.^9^,^11^,^20^,^21^,^30^–^33^ Our study was designed to examine the prevalence and distribution of four enteric viruses causing hospitalization in children attending two defined healthcare centers in one rural and one urban location in southern Vietnam. We found a substantial proportion of diarrhea to be caused by viral pathogens and an overall dominance of group A rotavirus. Our data suggest that, although this study represents a 1-month long snapshot of acute childhood diarrhea, viral pathogens are predominant etiological agents of acute childhood gastroenteritis in this location, a view that concurs with previous studies.^9^,^11^,^20^,^30^ We additionally show a variable distribution of enteric viruses in children hospitalized with acute watery diarrhea in distinct urban and rural locations in southern Vietnam, suggesting the circulation of different pathogens and corresponding differential infection risks.

Our findings show that group A rotaviruses were the predominant viral cause of diarrheal disease in children in both sampling locations. Within the group A rotavirus-positive samples, we identified extensive genetic diversity despite the limited temporal distribution and relatively small number of samples. Such dramatic diversity within the G1 group is consistent with multiple introductions of the G1 genotype to the region. Furthermore, only one of three G1 lineages showed a relationship to rotavirus sequences from HCMC in 2002–2005, again supporting on-going strain introduction with limited *in situ* evolution.^29^ Of all typeable P strains, P[8] predominated, with a comparable prevalence with North America, Australia, and Europe but higher prevalence than the prevalence previously reported in Vietnam.^9^–^11^,^14^,^20^ Again, phylogenetic analysis indicates that the extensive heterogeneity observed within the P[8] sequences is likely caused by multiple strain introduction rather than clonal expansion.

We identified a differential distribution of viral diarrheal pathogens between the urban and rural locations. We note a significantly higher prevalence of rotavirus genotype G12 in the rural location compared with the urban location. This study is the first to report a rotavirus genotype G12 in Vietnam, and since the primary detection in the Philippines in 1987, it has become increasingly prevalent worldwide.^34^ Our high detection rate (28.3% of all G types in DT) of this variant highlights the capacity of this genotype to spread and become fixed in a local population, and it has direct implications for rotavirus vaccination, because protective immunity of two available rotavirus vaccines (RotaRix and RotaTeq) against G12 genotype is currently undetermined. However, a high evolutionary rate of the VP7 gene (1.66 × 10^−3^ substitutions/site per year) suggests that the introduction of either vaccine may impose a selective pressure on circulating strains and accelerate evolutionary rates, facilitating the emergence and rapid spread of variants.^35^ Such factors emphasize the need for on-going modification and development of rotavirus vaccines and continued surveillance for genotype circulation.

Secondary data supporting a differential distribution of enteric viruses between urban and rural settings is the distribution of norovirus in the two locations. Norovirus was the second most commonly identified virus in the patients' stool samples, which is similar to global data.^36^ From the positive stool samples in HCMC, norovirus constituted 29% of positive sample compared with only 5.1% in DT. We suggest different epidemiological risk factors related to this organism in these locations, which necessitates additional investigation. Norovirus is highly contagious and is related to isolated outbreaks in developed countries.^37^ HCMC is more densely populated and has undergone a greater level of development with respect to the surrounding province, such as DT. Transmission and the corresponding exposure to this particular pathogen are likely to follow such a developmental change. In parallel to rotavirus infections, the majority of patients in which we detected norovirus in the stool samples was in the 7–12 months age group. This age group is the key age group for children with diarrheal infections and poses the greatest number of epidemiological questions. Decreased rates of all viral pathogens detected in the stool samples outside this key age group may be related to risk, exposure, healthcare-seeking behavior, maternal immunity, and natural postinfection immunity. Additional studies on the effect maternal antibody or immunity from previous exposure to norovirus should help guide vaccine development and usage within this age group.

Our study design does have several caveats; first, although the age distribution of enrolled patients was similar in both locations, such sampling was open to selection bias. However, we aimed to understand the distribution viral diarrheal pathogen and not determine risk factors; therefore, a limited period of sampling may bias the distribution of the various agents. Second, it is known that 5% of control healthy patients (defined as inpatients for non-infectious causes without diarrhea in the previous 2 weeks) between the ages of 3 months and 5 years can carry viral pathogens in their stool specimens, and additional sampling is necessary to understand the transmission dynamics and asymptomatic carriage of enteric viruses.^32^ We did find some dual viral infections (4.1%), but we were unable to elucidate the impact in pathogenesis and their clinical significance, which also may be an issue when other bacterial and parasitic agents are considered.

In conclusion, this report is the first report of rotavirus G12 in Vietnam, and we show a differential distribution of the major enteric viral pathogens and rotavirus genotypes causing childhood acute gastroenteritis in two distinct locations in southern Vietnam. We highlight the need for longitudinal research of enteric viruses in this location and continued monitoring of circulating rotavirus strains for effective prevention and vaccination strategies.

## Figures and Tables

**Table 1:** Enzyme immunoassay detection of four viral pathogens in stool samples from children with acute diarrhea in Ho Chi Minh City and Dong Thap

Viral assay	Number of positive samples in Ho Chi Minh City (%)*	Number of positive samples in Dong Thap (%)†
Group A rotavirus‡	75 (29.8)	72 (65.5)
Norovirus	34 (13.5)	4 (3.6)
Adenovirus	6 (2.4)	3 (2.7)
Astrovirus	7 (2.8)	2 (1.8)
Multiple positive	5 (4.3)	3 (2.7)
Total viruses detected	122	81
Total positive samples	117 (46.4)	78 (73.6)

* *N* = 252.

† *N* = 110.

‡ Equivocal results were found in two samples from Ho Chi Minh City.
